# Supplementary material for: STAT3 promotes IFNγ/TNFα‐induced muscle wasting in an NF‐κB‐dependent and IL‐6‐independent manner
Source: EMBO Mol Med. 2017 Mar 6;9(5):622–37. doi: 10.15252/emmm.201607052 (PMC5412921; doi:10.15252/emmm.201607052)
Supplement: Supplementary file 1 — Appendix [file EMMM-9-622-s001.pdf]

Appendix Ma et al. (EMM-2016-07052)

Appendix Supplementary Methods

Appendix References

Appendix Supplementary Figure S1 to S5 with figure legends

Appendix Supplementary Methods

Primary myoblast cell culture

The protocol used for skeletal muscle satellite cell isolation was previously described (Demoule, Divangahi et al., 2005). Briefly, the tibialis anterior muscle was dissected out and digested in 0.2% collagenase for 1h 45m. The digested tissue was gently pipetted to liberate single fibers were plated onto Matrigel-coated flasks. After four days of incubation at 37°C and 5% CO_2 in plating medium (10% horse serum, 0.5% chick embryo extract in DMEM), the cells began sub-culturing every two days. Proliferating myoblasts were maintained in proliferation medium (20% FBS, 10% HS, 1% CEE in DMEM). To differentiate, the cells were switched to differentiation media (2% FBS, 10% HS, 0.5% CEE in DMEM) for four days then to DMEM with 2% HS for one day before treatment.

Appendix References

Demoule A, Divangahi M, Danialou G, Gvozdic D, Larkin G, Bao W, Petrof BJ (2005) Expression and regulation of CC class chemokines in the dystrophic (mdx) diaphragm. Am J Respir Cell Mol Biol 33: 178-85

Supplemental Figures

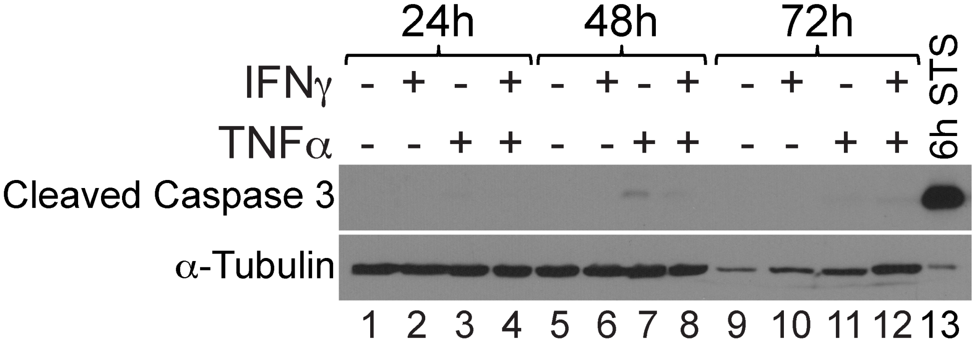

<u>Appendix Figure S1</u>: ***The treatment of C2C12 myotubes with both TNFα and IFNγ for 72h does not trigger caspase 3 cleavage.*** Total cell extracts were prepared from C2C12 myotubes treated with IFNγ alone, TNFα alone, or both IFNγ and TNFα for the indicated amount of time. Extracts from C2C12 treated with staurosporine for 6 hrs was also prepared and used as a positive control. Western blotting experiment was performed using these extracts with antibody specific for caspase-3 cleavage product and anti-α-tubulin antibody. The blot shown is a representative of two independent experiments.

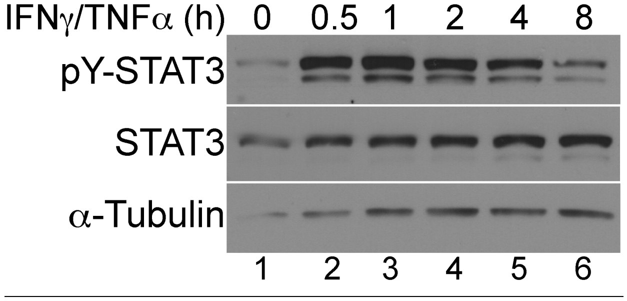

<u>Appendix Figure S2</u>: ***IFNγ/TNFα rapidly triggers the phosphorylation of STAT3 on its Y705 residue (pY-STAT3) in differentiated primary muscle cells***. Total cell extracts from differentiated primary muscle cells stimulated with or without IFNγ/TNFα for 0h to 8h were used for Western blot analysis with antibodies against pY-STAT3, total STAT3, and α-tubulin. The blot shown is a representative of three independent experiments.

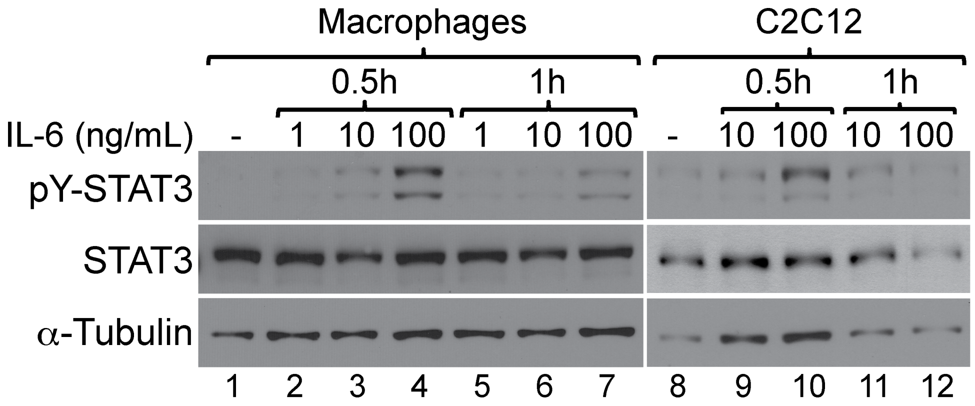

<u>Appendix Figure S3</u>**: *IL-6 induced phosphorylation of STAT3 in both Macrophages and C2C12 cells occurs in a dose-dependent manner.*** Total cell extract from macrophages or C2C12 myotubes were treated with 10 or 100 ng/mL of recombinant mouse IL-6 for the indicated length of time were used for Western blot analysis with antibodies against pY-STAT3, total STAT3, and α-tubulin. The 100ng/mL dose of rIL-6 induced pY-STAT3 in both macrophages and C2C12 myotubes while the 10 ng/mL dose was only able to induce pY-STAT3 in macrophages. The blot shown is a representative of two independent experiments.

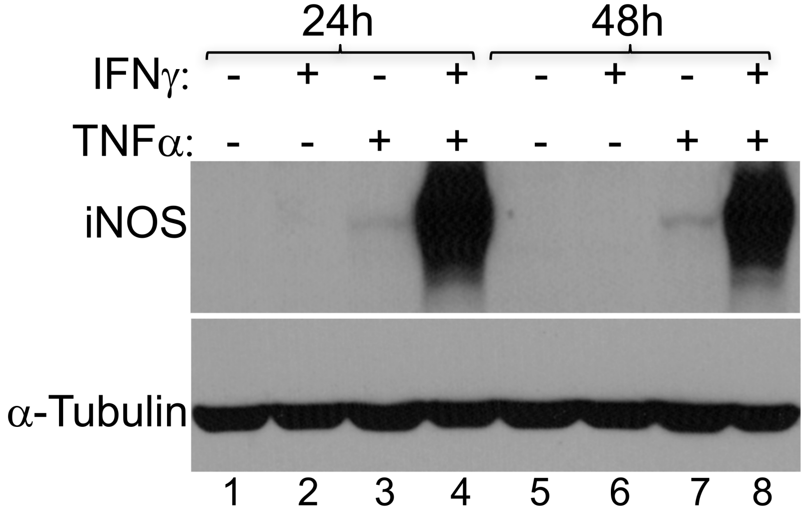

<u>Appendix Figure S4 (related to Figure 4)</u>**: *IFNγ and TNFα synergistically induce the expression of iNOS in C2C12 muscle fibers.*** Total cell extracts were prepared from C2C12 myotubes treated with IFNγ/TNFα for the indicated amount of time. These extracts were used for Western blot analysis with antibodies against iNOS and α-tubulin. The blot shown is a representative of two independent experiments.

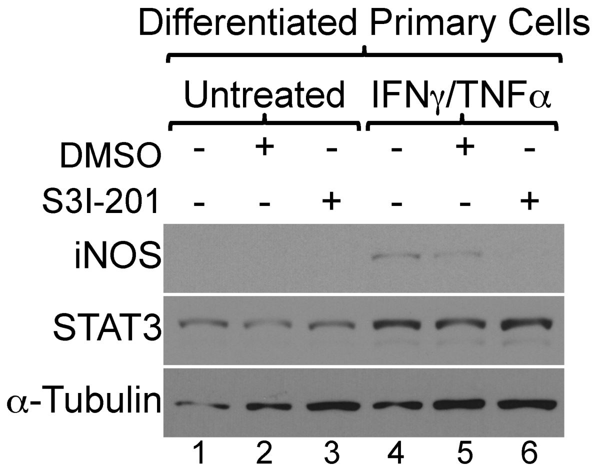

<u>**Appendix Figure S5**</u> <u>(related to Figure 4)</u>**:** ***Inhibition of STAT3 activity prevents IFNγ/TNFα -induced iNOS expression in differentiated primary muscle cells.*** Total cell extracts were prepared from differentiated primary muscle cells treated with IFNγ/TNFα in the presence or absence of the STAT3 inhibitor S3I-201 > 3 days. Western blot analysis indicated that inhibiting STAT3 in primary cells blocks IFNγ/TNFα-induced iNOS expression. The blot shown is a representative of three independent experiments.
