# Supplementary figures and images for: STAT3 promotes IFNγ/TNFα‐induced muscle wasting in an NF‐κB‐dependent and IL‐6‐independent manner
Source: EMBO Mol Med. 2017 Mar 6;9(5):622–37. doi: 10.15252/emmm.201607052 (PMC5412921; doi:10.15252/emmm.201607052)

## Slide 1
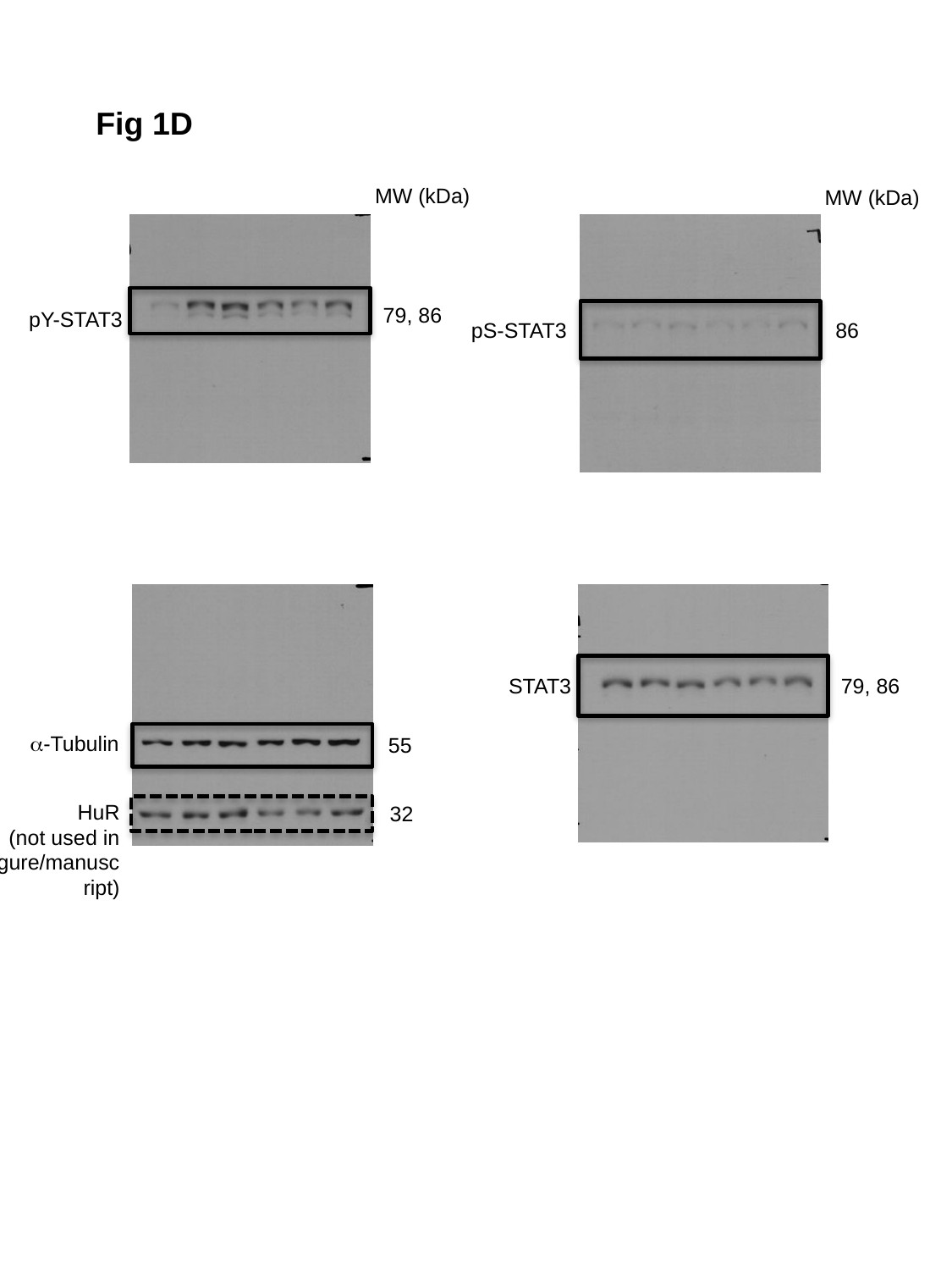

Fig 1D
MW (kDa)
MW (kDa)
79, 86
pY-STAT3
pS-STAT3
86
STAT3
79, 86
a-Tubulin
55
HuR
 (not used in figure/manuscript)
32

Supplement: Supplementary file 3 — Source Data for Figure 1 [file EMMM-9-622-s002.pptx]

## Slide 1
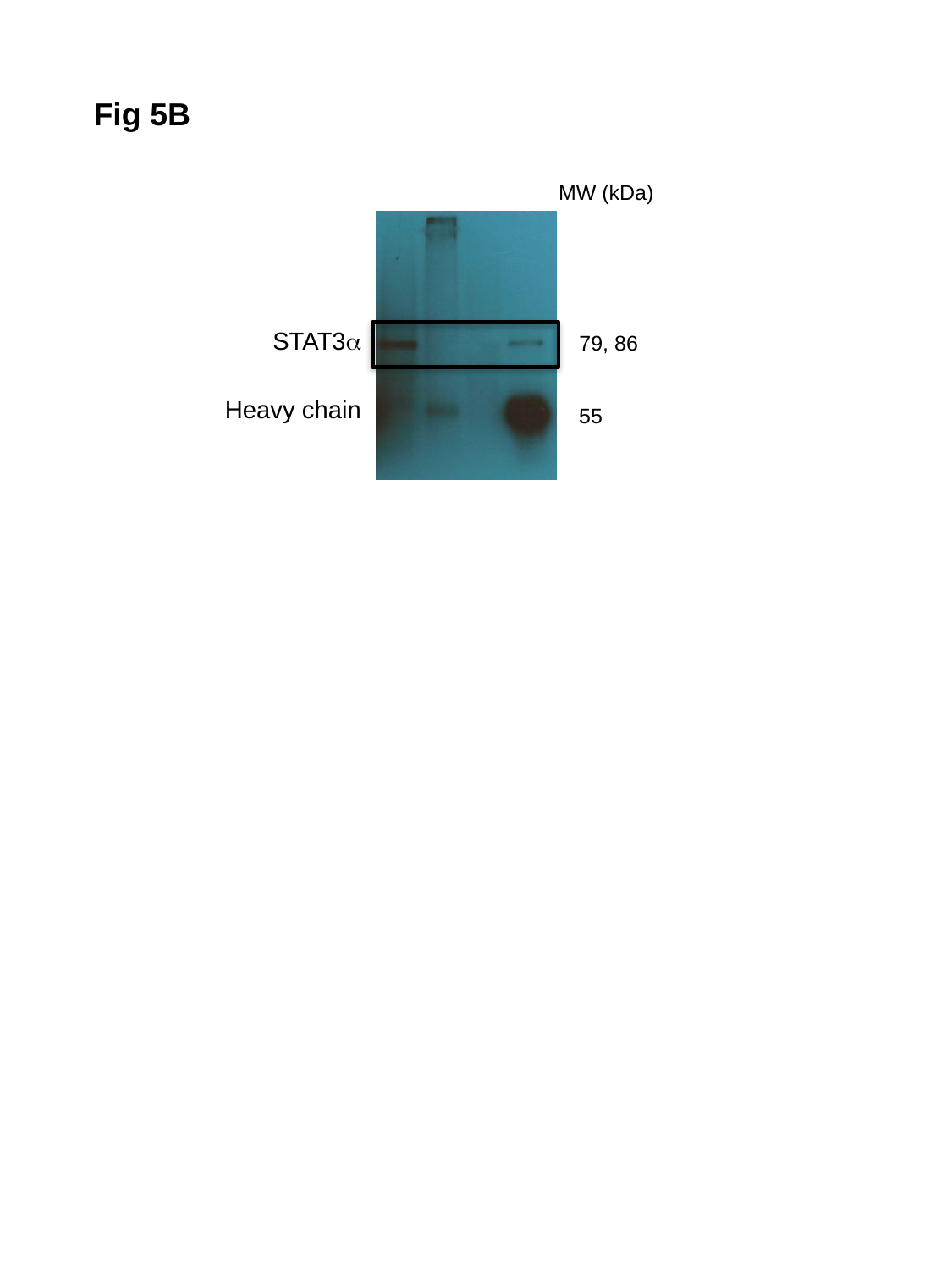

Fig 5B
MW (kDa)
STAT3a
79, 86
Heavy chain
55

## Slide 2
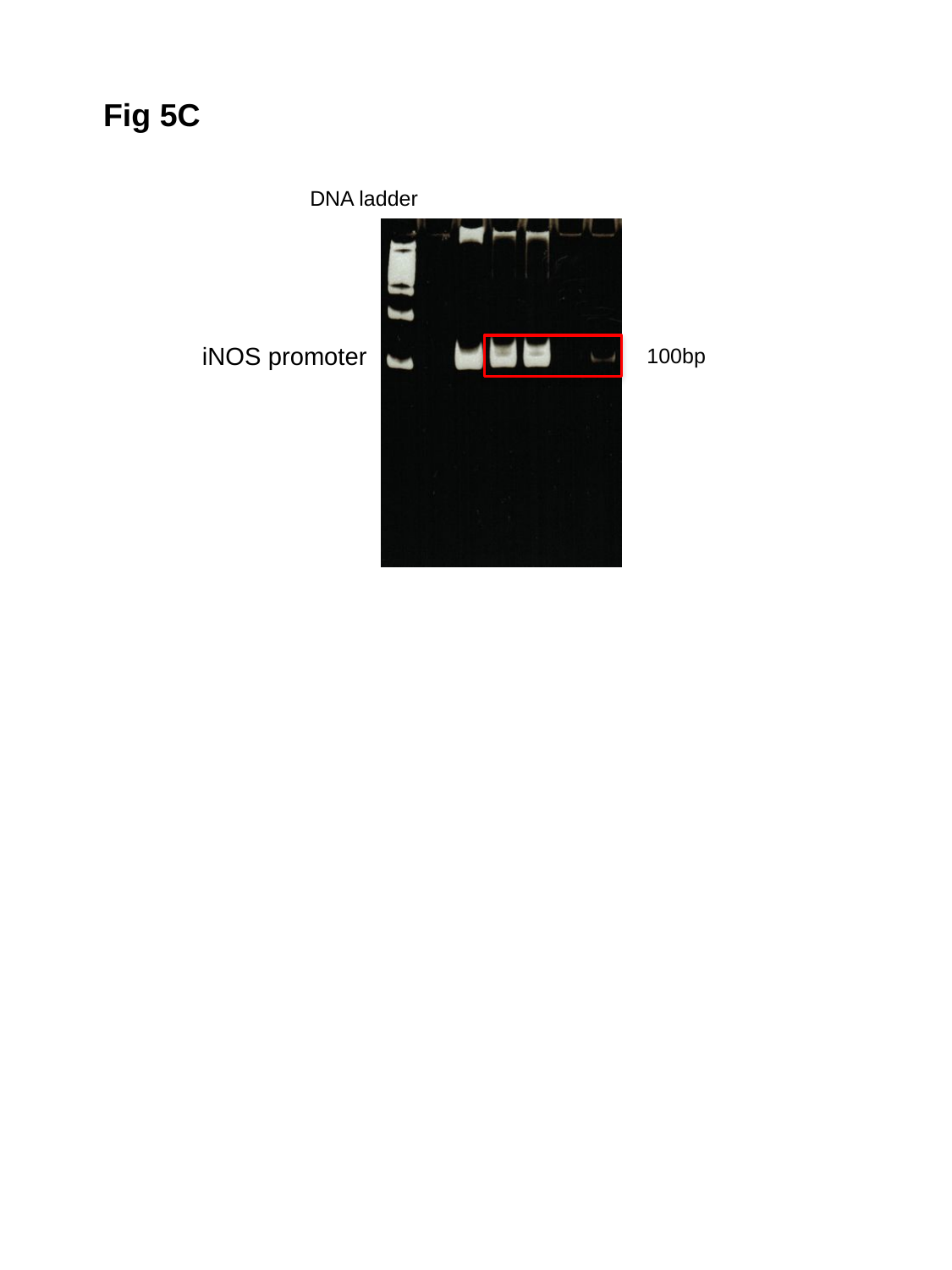

Fig 5C
DNA ladder
iNOS promoter
100bp

Supplement: Supplementary file 7 — Source Data for Figure 5 [file EMMM-9-622-s006.pptx]

## Slide 1
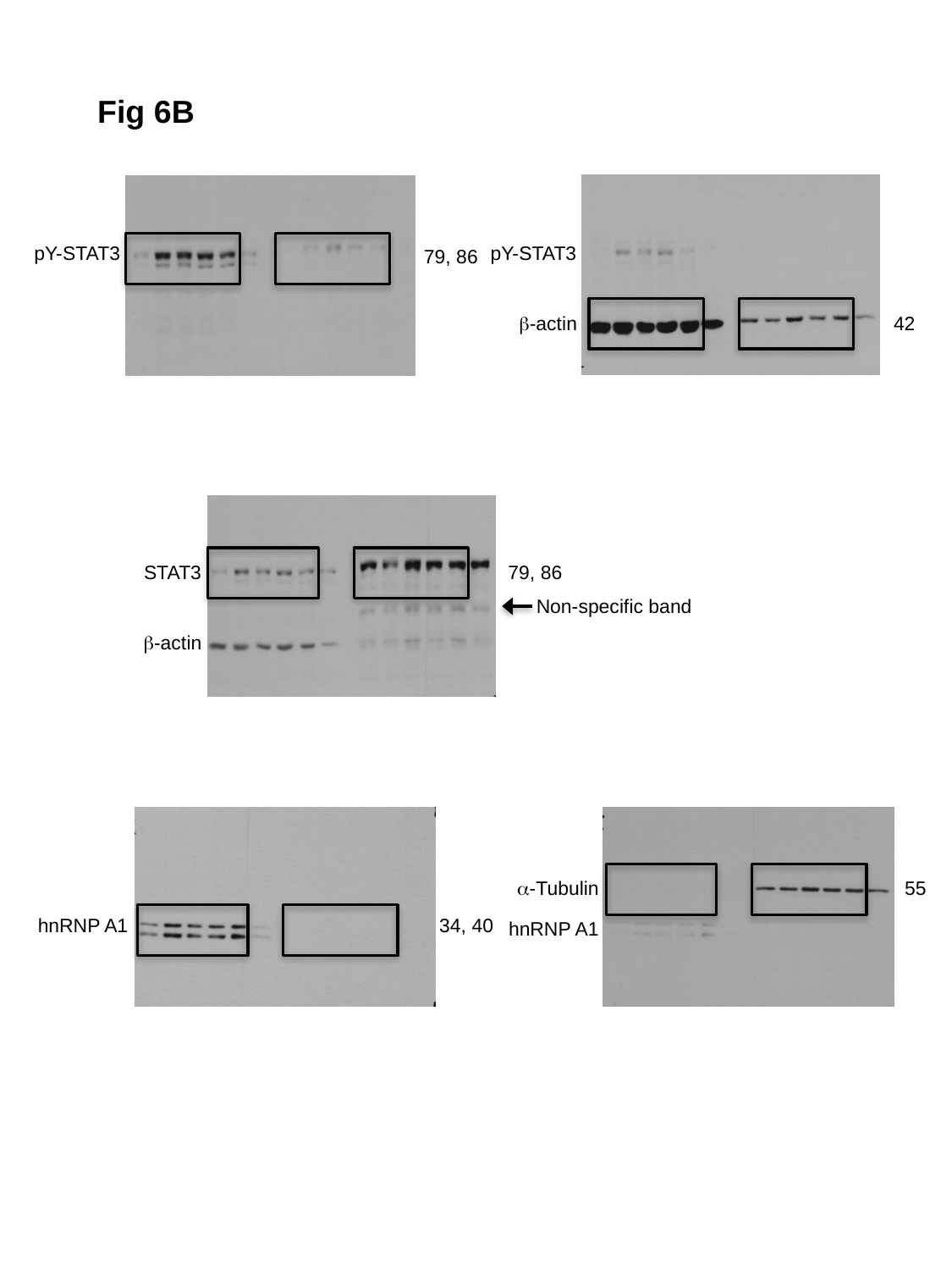

Fig 6B
pY-STAT3
pY-STAT3
79, 86
b-actin
42
STAT3
79, 86
Non-specific band
b-actin
a-Tubulin
55
hnRNP A1
34, 40
hnRNP A1

Supplement: Supplementary file 8 — Source Data for Figure 6 [file EMMM-9-622-s007.pptx]
