# Supplementary material for: STAT3 promotes IFNγ/TNFα‐induced muscle wasting in an NF‐κB‐dependent and IL‐6‐independent manner
Source: EMBO Mol Med. 2017 Mar 6;9(5):622–37. doi: 10.15252/emmm.201607052 (PMC5412921; doi:10.15252/emmm.201607052)
Supplement: Supplementary file 4 — Source Data for Figure 2 [file EMMM-9-622-s003.pptx]

## Slide 1
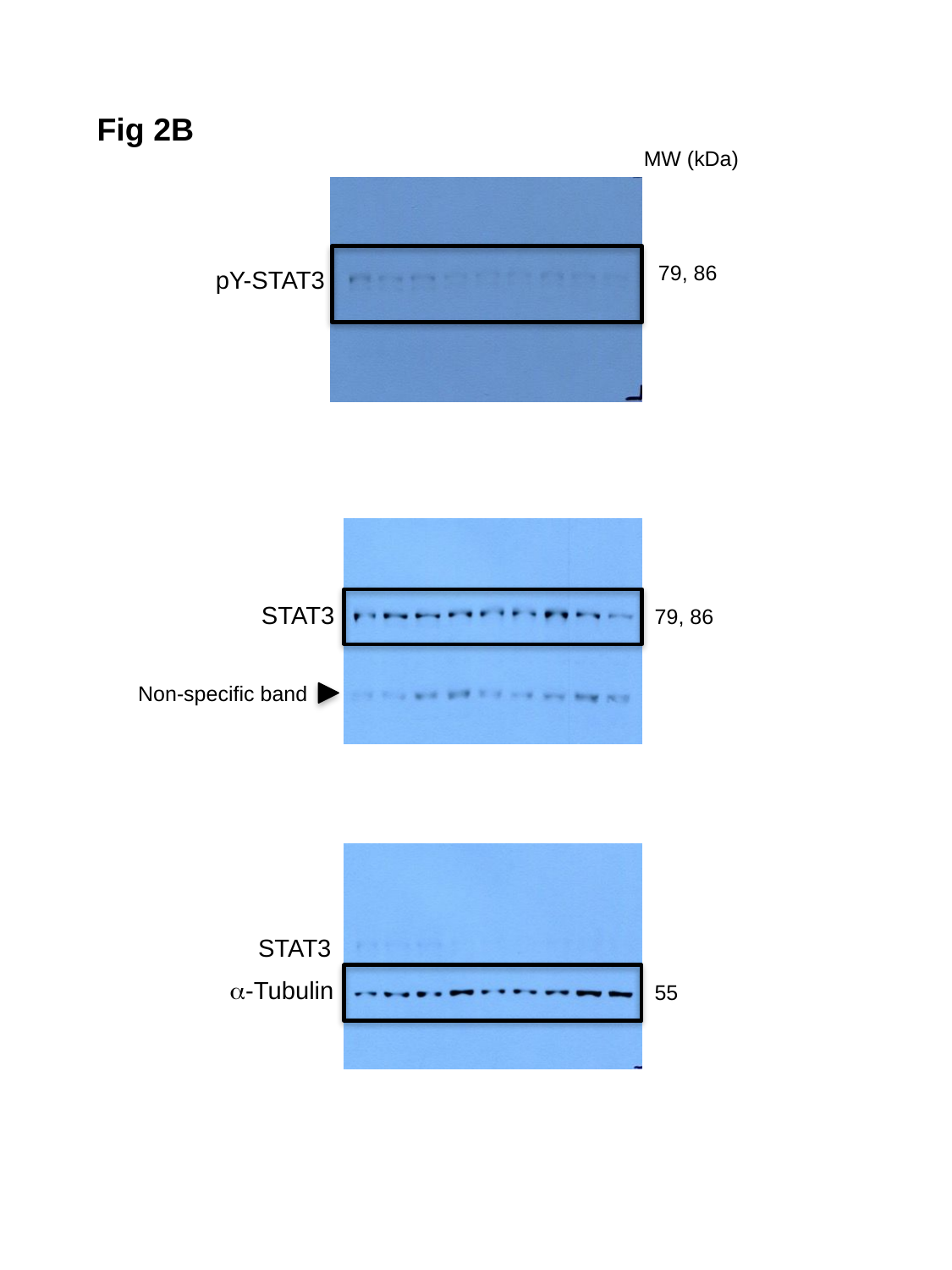

Fig 2B
MW (kDa)
79, 86
pY-STAT3
STAT3
79, 86
Non-specific band
STAT3
a-Tubulin
55

## Slide 2
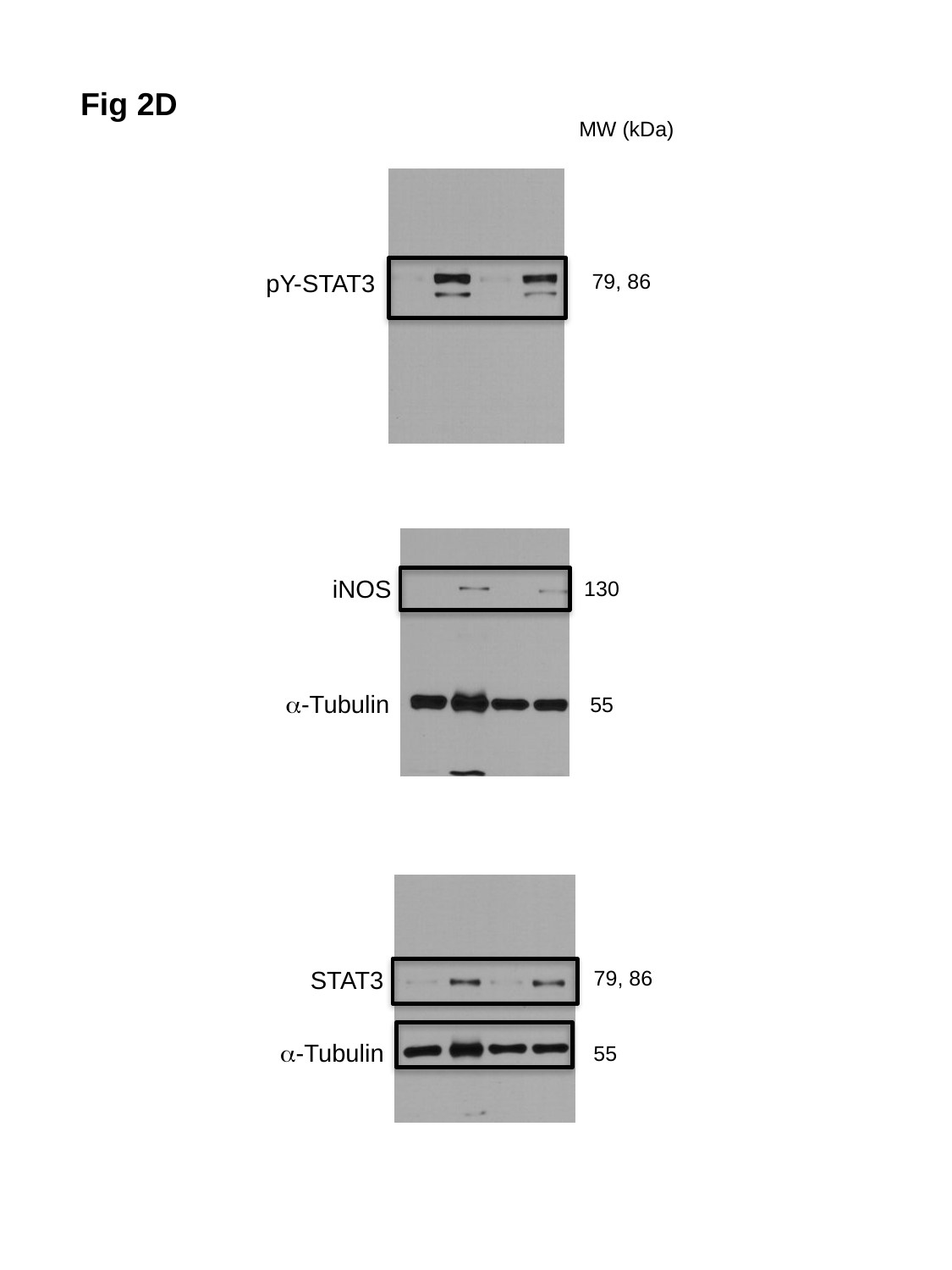

Fig 2D
MW (kDa)
pY-STAT3
79, 86
iNOS
130
a-Tubulin
55
STAT3
79, 86
a-Tubulin
55
