# Supplementary material for: STAT3 promotes IFNγ/TNFα‐induced muscle wasting in an NF‐κB‐dependent and IL‐6‐independent manner
Source: EMBO Mol Med. 2017 Mar 6;9(5):622–37. doi: 10.15252/emmm.201607052 (PMC5412921; doi:10.15252/emmm.201607052)
Supplement: Supplementary file 5 — Source Data for Figure 3 [file EMMM-9-622-s004.pptx]

## Slide 1
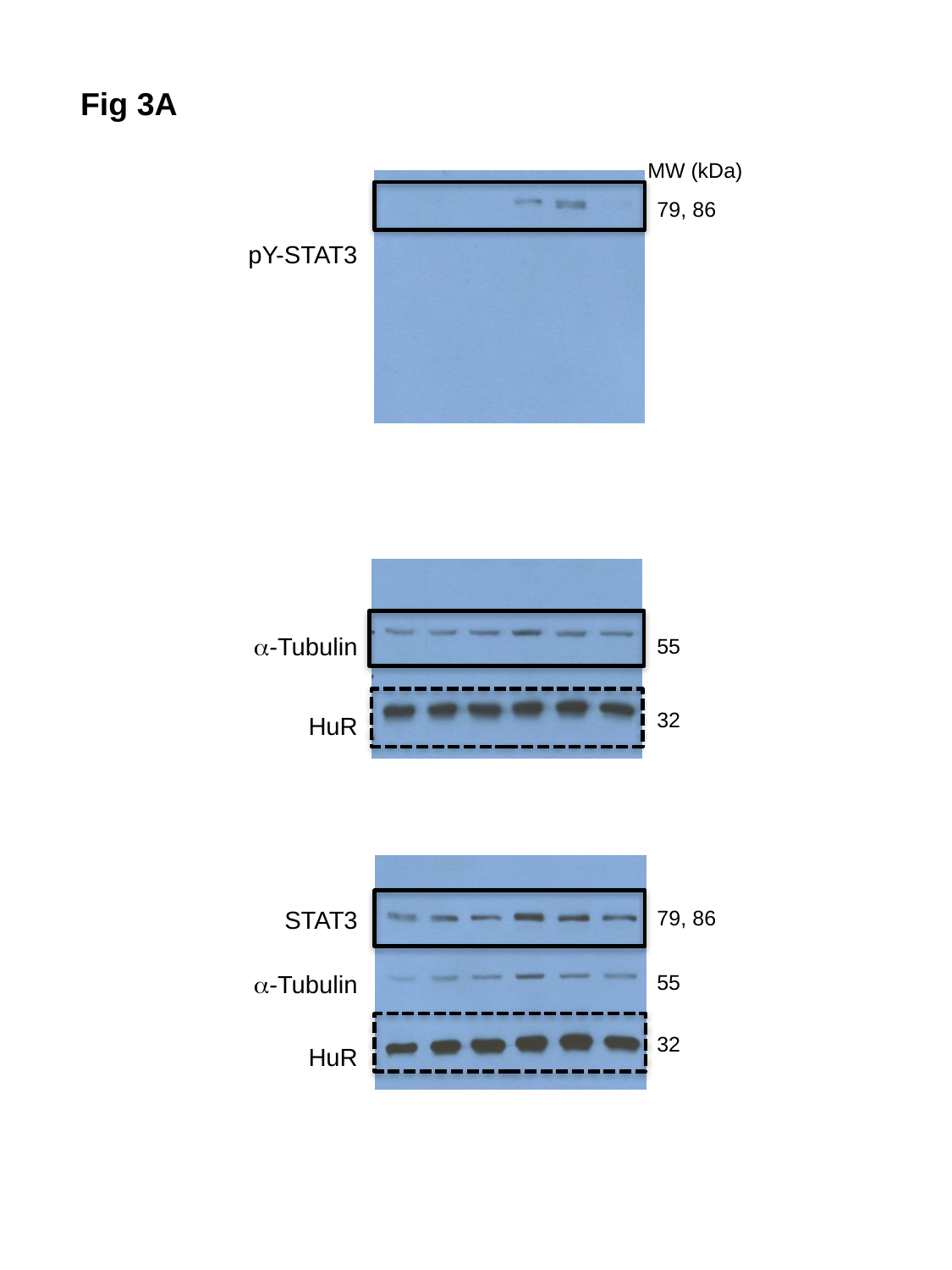

Fig 3A
MW (kDa)
79, 86
pY-STAT3
a-Tubulin
55
32
HuR
STAT3
79, 86
a-Tubulin
55
32
HuR

## Slide 2
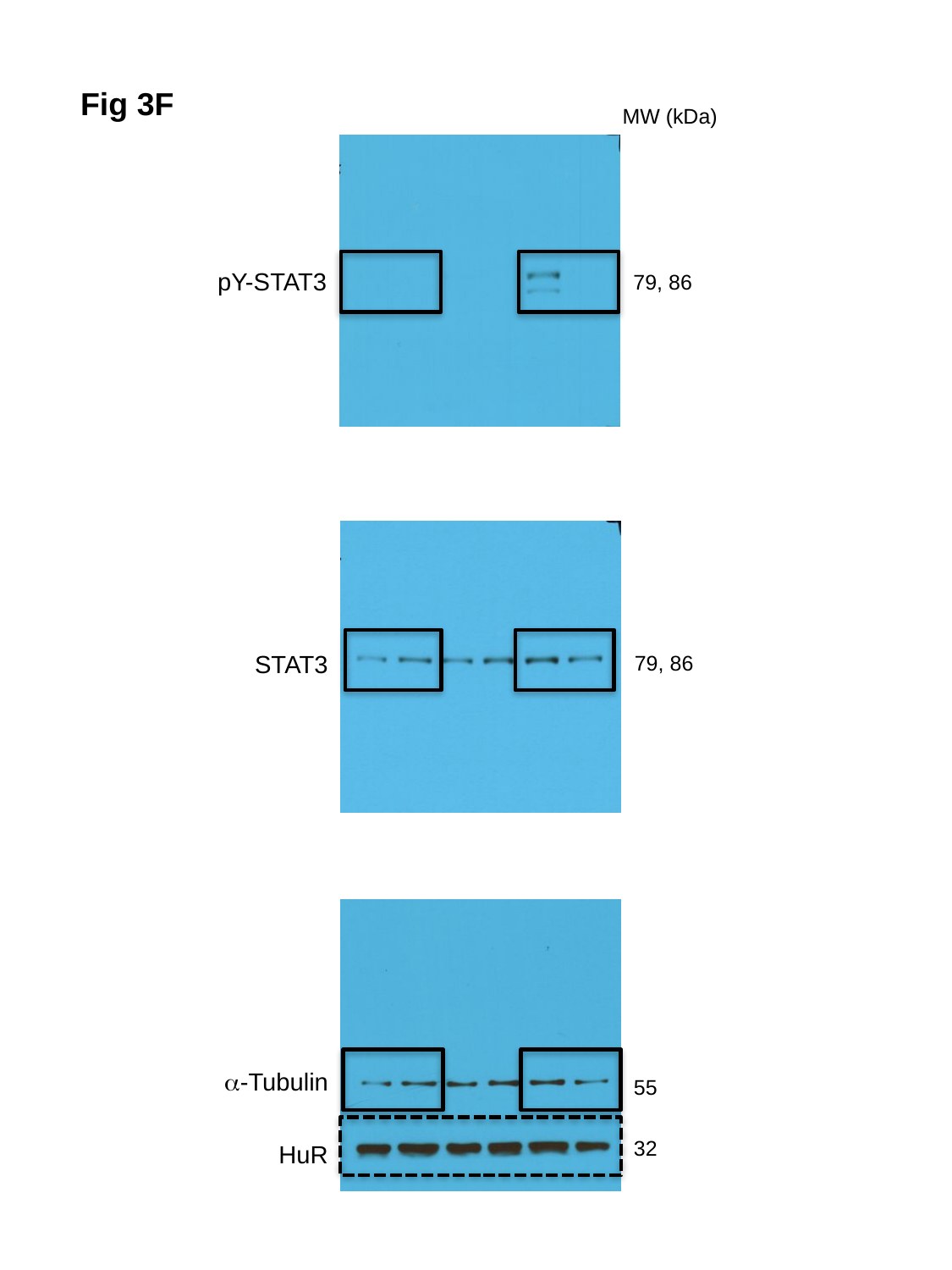

Fig 3F
MW (kDa)
pY-STAT3
79, 86
STAT3
79, 86
a-Tubulin
55
32
HuR
