# Supplementary material for: STAT3 promotes IFNγ/TNFα‐induced muscle wasting in an NF‐κB‐dependent and IL‐6‐independent manner
Source: EMBO Mol Med. 2017 Mar 6;9(5):622–37. doi: 10.15252/emmm.201607052 (PMC5412921; doi:10.15252/emmm.201607052)
Supplement: Supplementary file 6 — Source Data for Figure 4 [file EMMM-9-622-s005.pptx]

## Slide 1
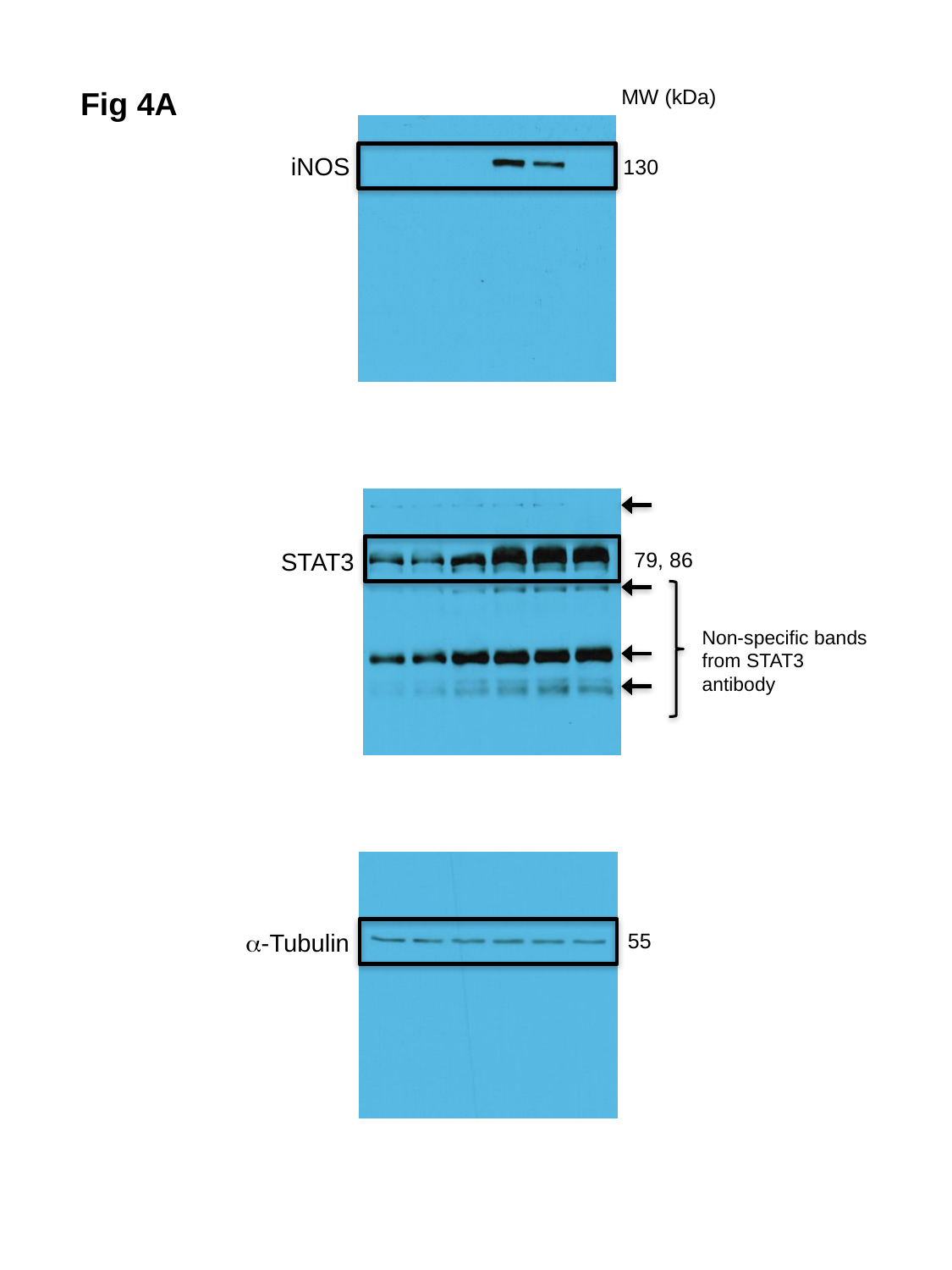

MW (kDa)
Fig 4A
iNOS
130
79, 86
STAT3
Non-specific bands from STAT3 antibody
a-Tubulin
55

## Slide 2
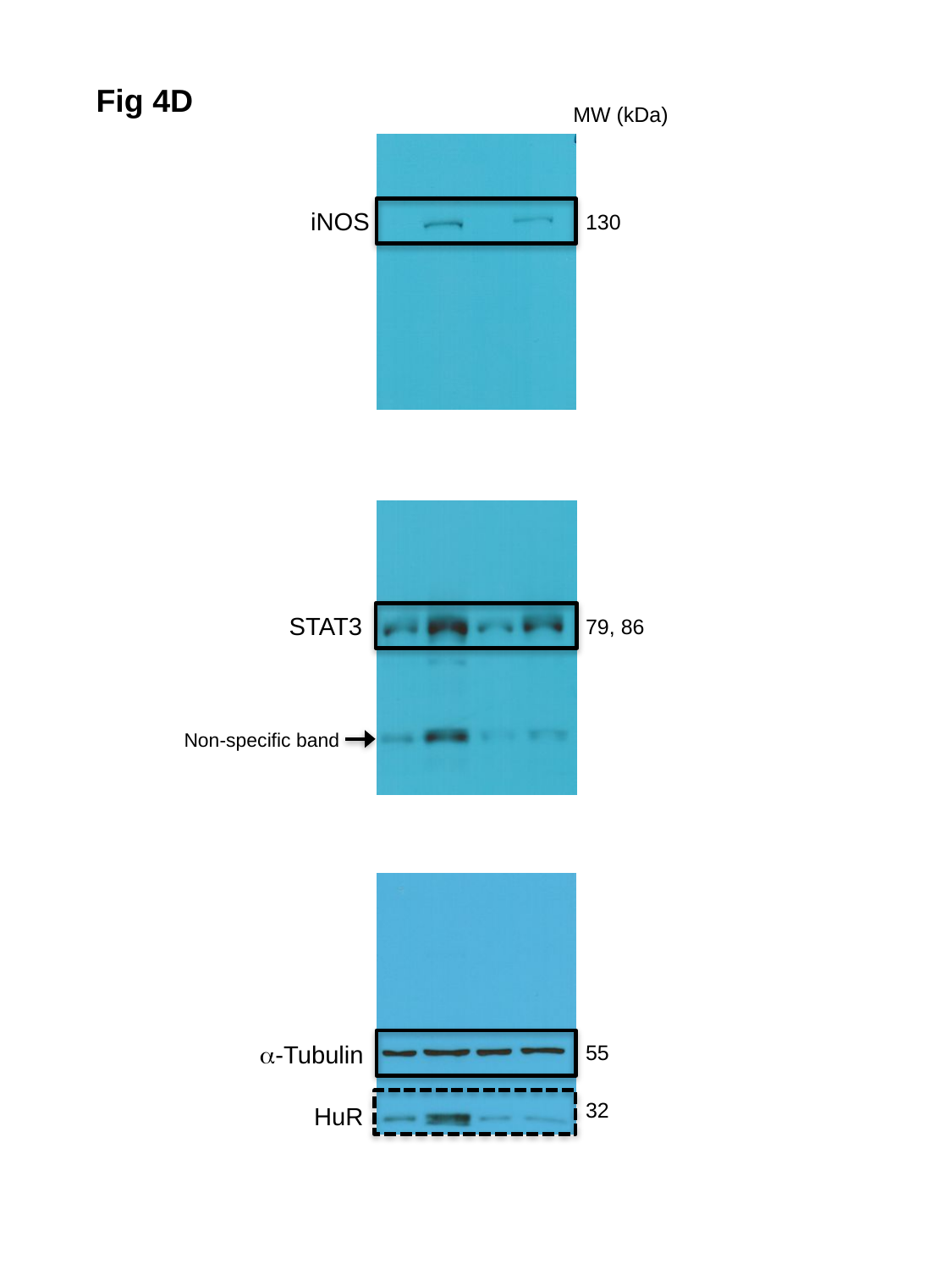

Fig 4D
MW (kDa)
iNOS
130
STAT3
79, 86
Non-specific band
a-Tubulin
55
32
HuR

## Slide 3
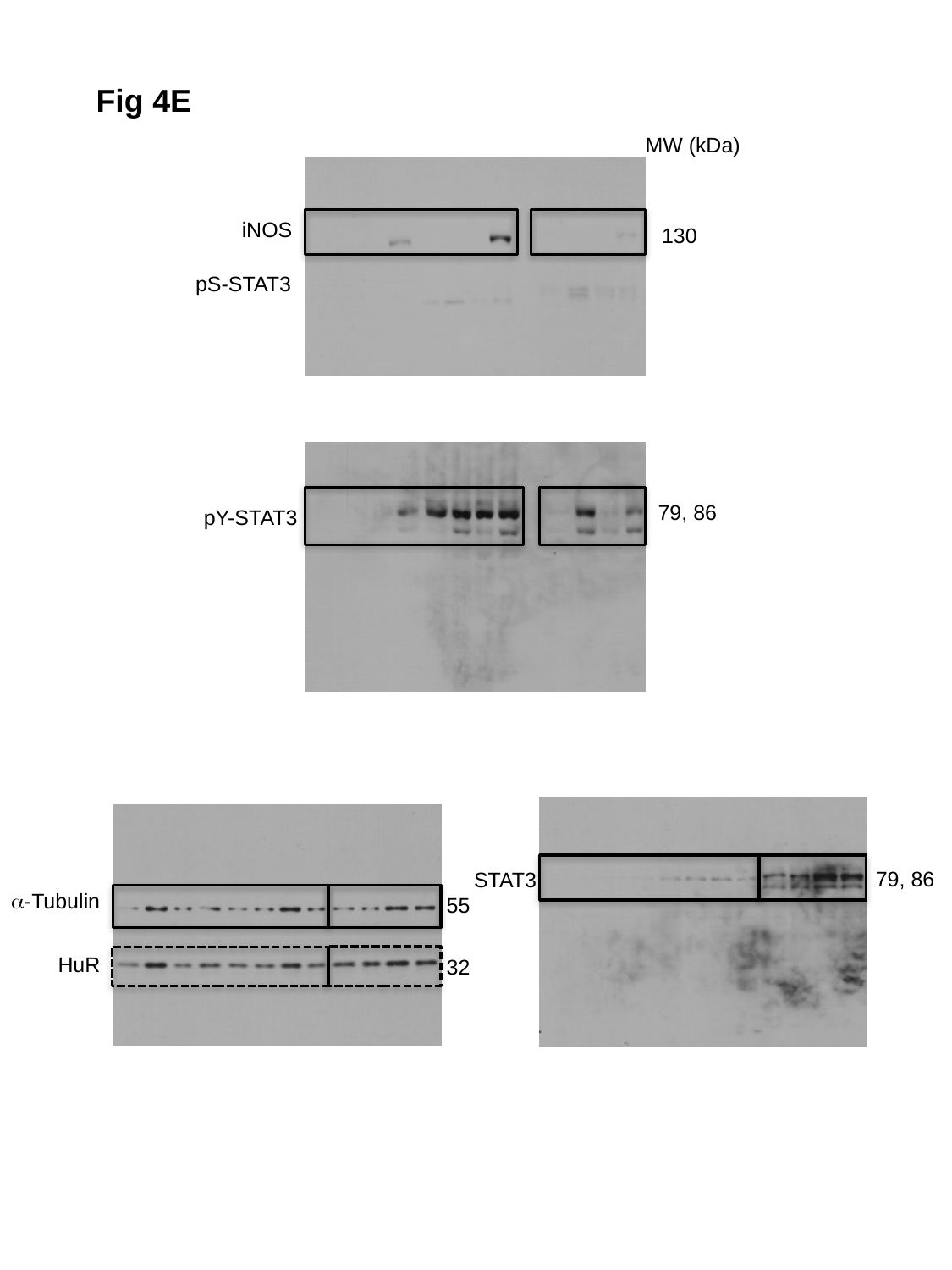

Fig 4E
MW (kDa)
iNOS
130
pS-STAT3
79, 86
pY-STAT3
79, 86
STAT3
a-Tubulin
55
HuR
32

## Slide 4
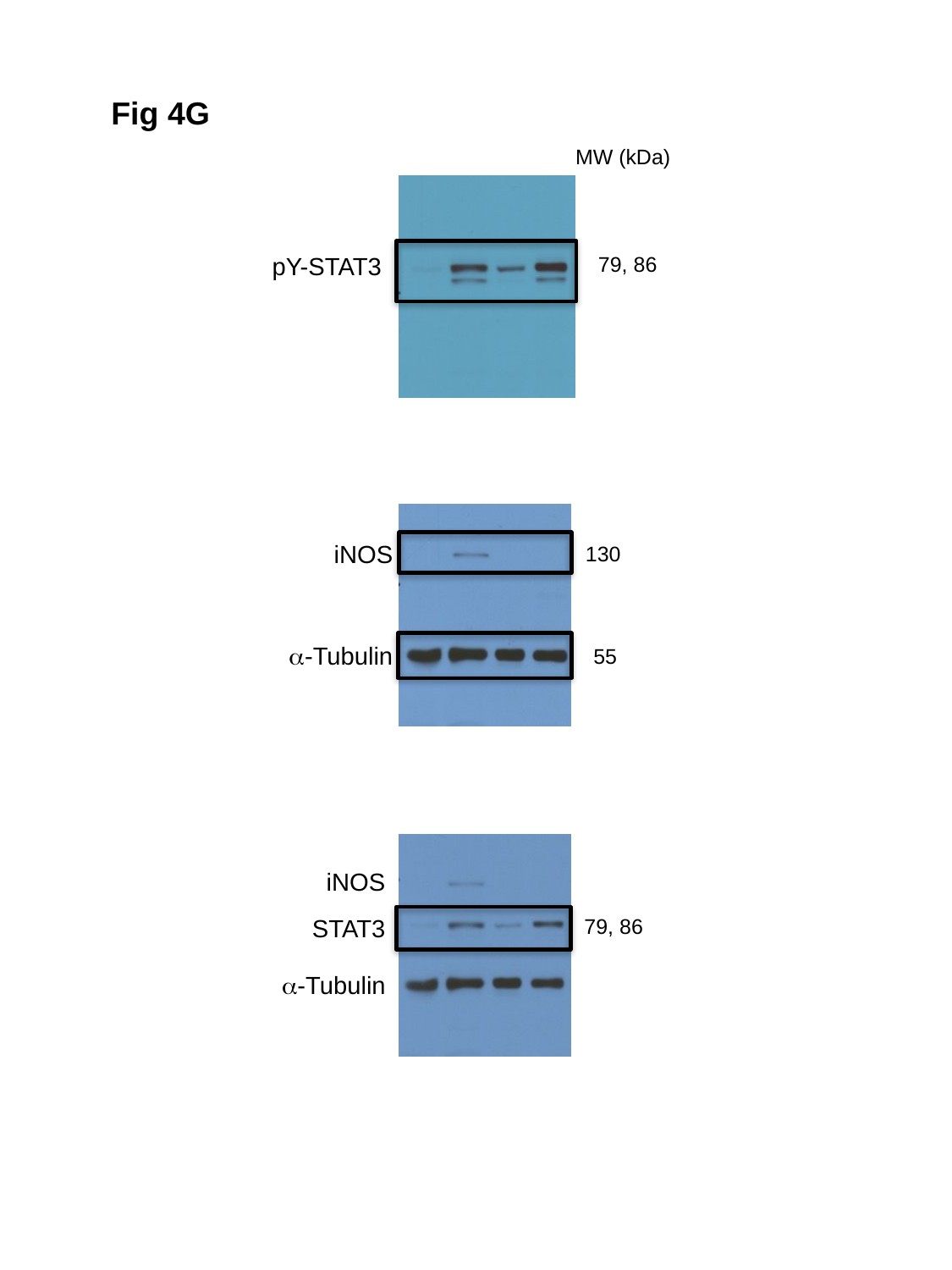

Fig 4G
MW (kDa)
pY-STAT3
79, 86
iNOS
130
a-Tubulin
55
iNOS
STAT3
79, 86
a-Tubulin
