# Supplementary material for: STAT3 promotes IFNγ/TNFα‐induced muscle wasting in an NF‐κB‐dependent and IL‐6‐independent manner
Source: EMBO Mol Med. 2017 Mar 6;9(5):622–37. doi: 10.15252/emmm.201607052 (PMC5412921; doi:10.15252/emmm.201607052)
Supplement: Supplementary file 9 — Source Data for Figure 7 [file EMMM-9-622-s008.pptx]

## Slide 1
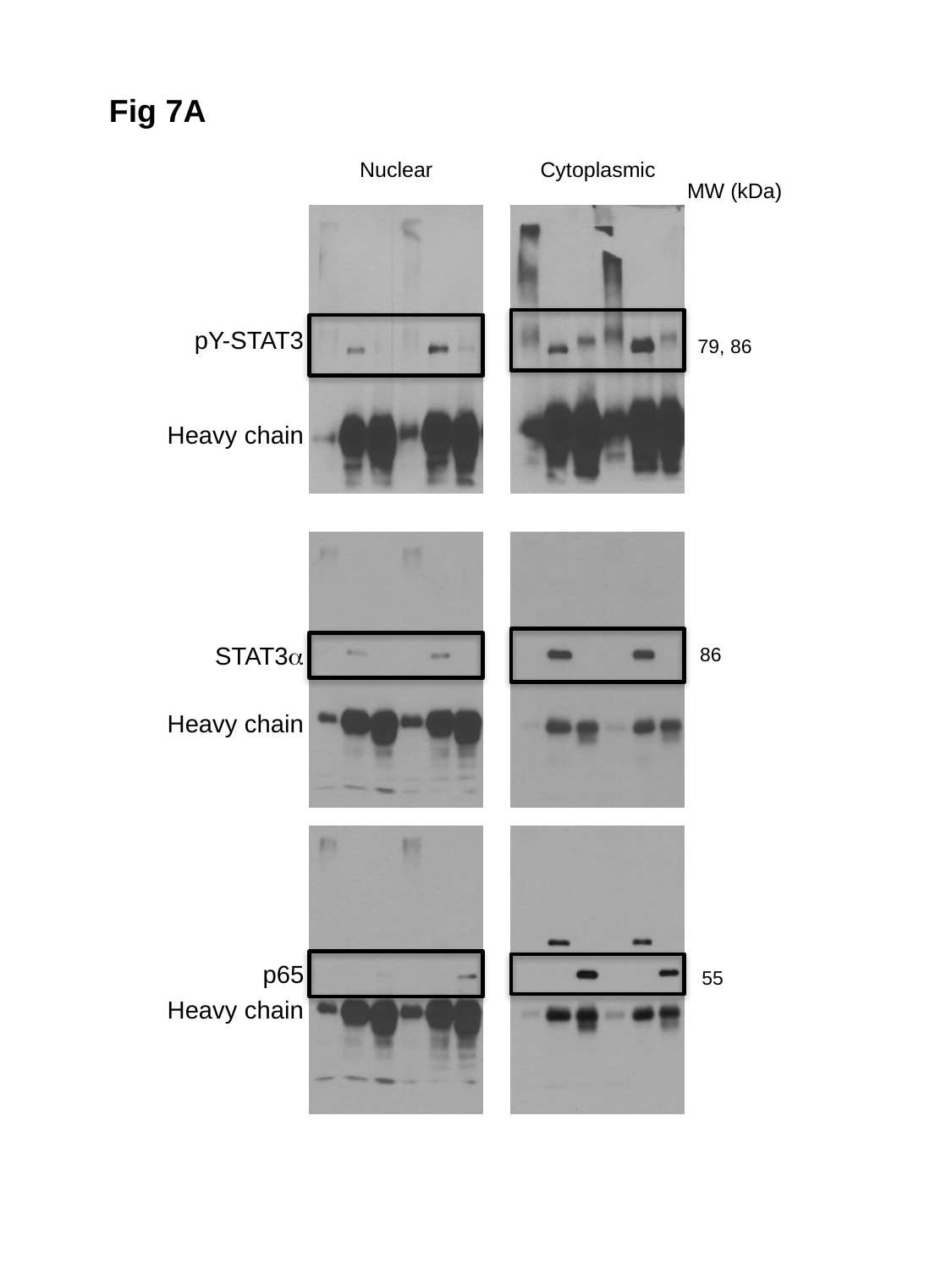

Fig 7A
Nuclear
Cytoplasmic
MW (kDa)
pY-STAT3
79, 86
Heavy chain
STAT3a
86
Heavy chain
p65
55
Heavy chain

## Slide 2
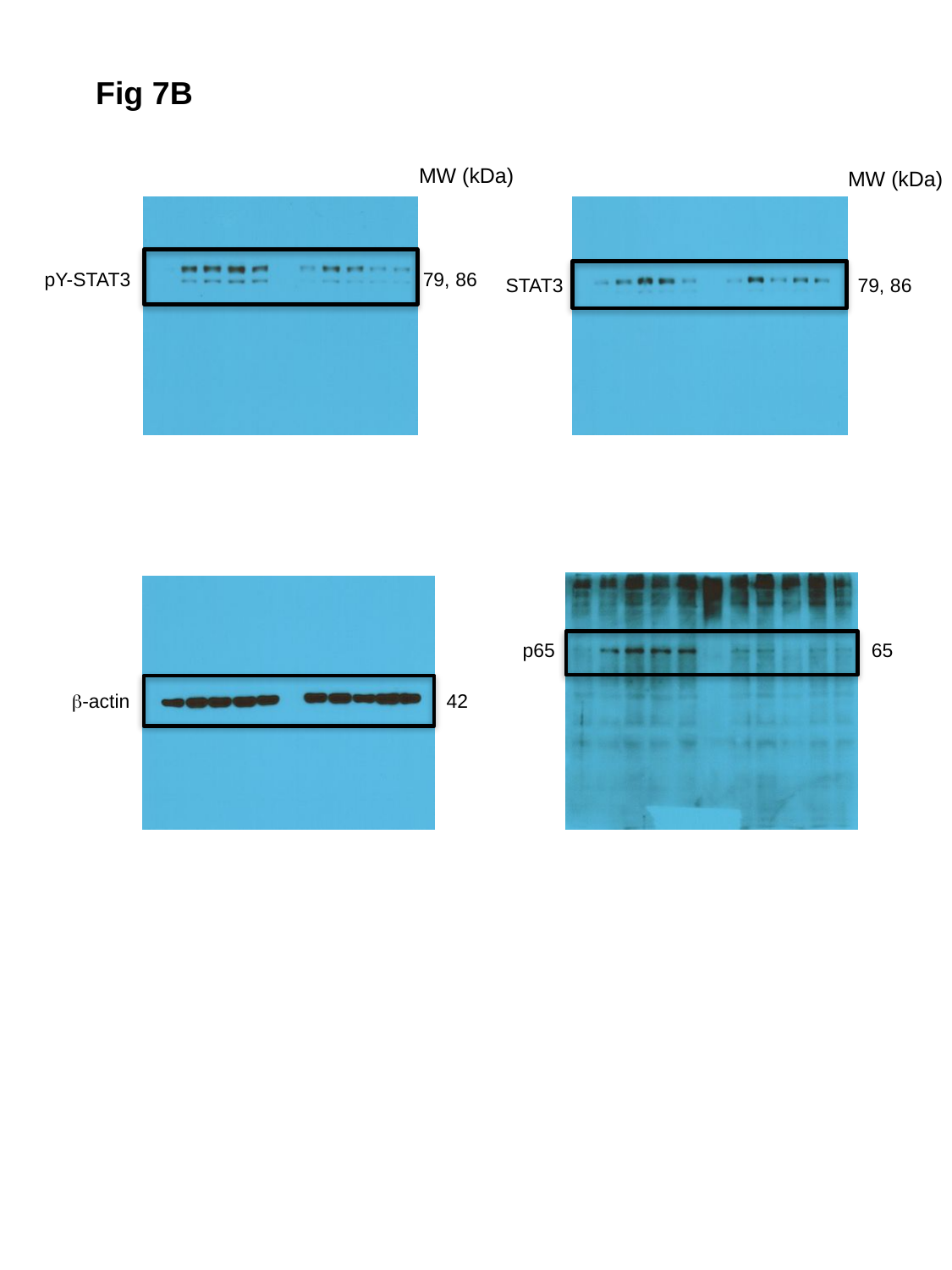

Fig 7B
MW (kDa)
MW (kDa)
pY-STAT3
79, 86
STAT3
79, 86
p65
65
b-actin
42

## Slide 3
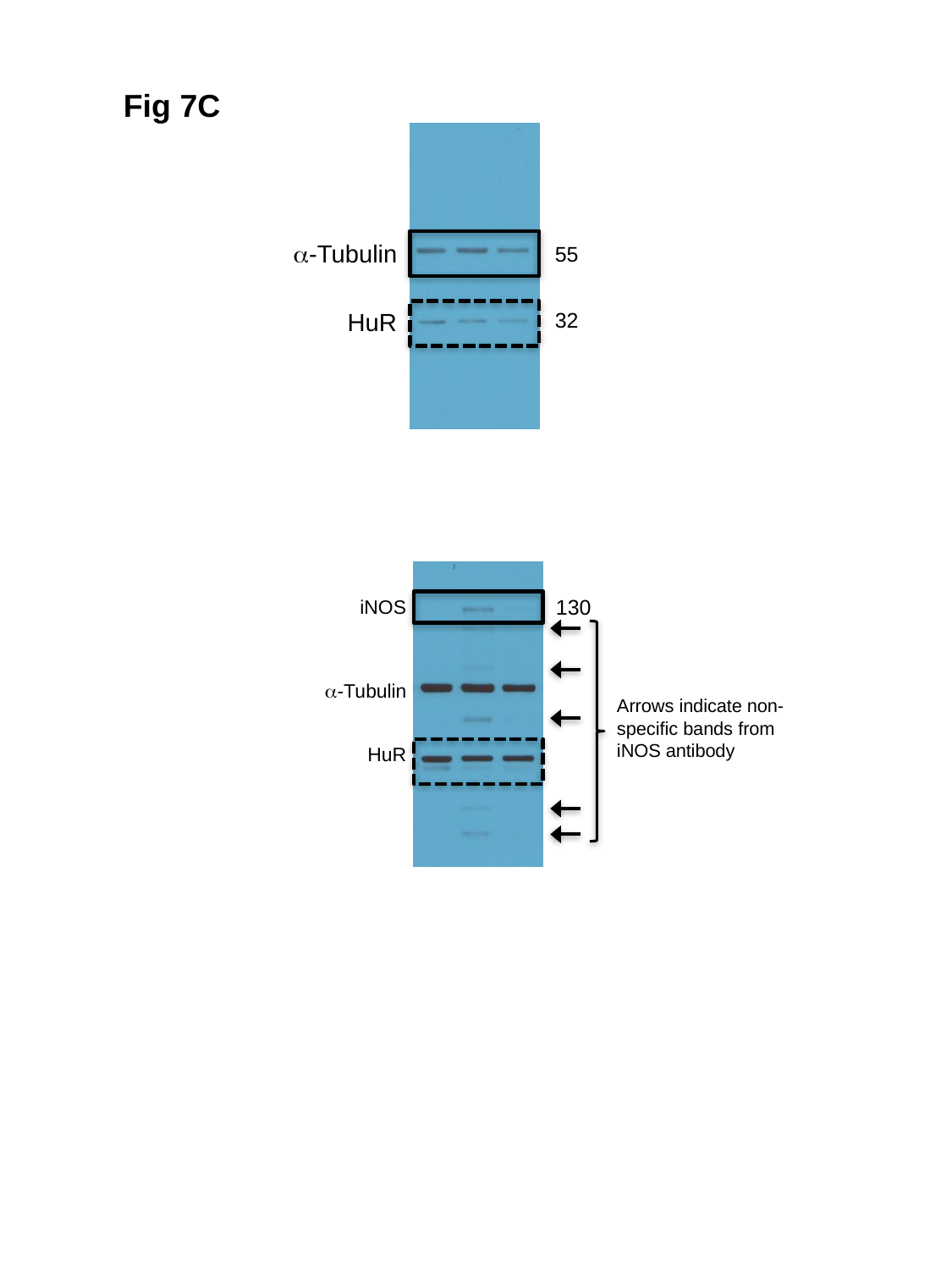

Fig 7C
a-Tubulin
55
HuR
32
130
iNOS
a-Tubulin
Arrows indicate non-specific bands from iNOS antibody
HuR
